# Individualized induction chemotherapy by pre-treatment plasma Epstein-Barr viral DNA in advanced nasopharyngeal carcinoma

**DOI:** 10.1186/s12885-018-5177-9

**Published:** 2018-12-19

**Authors:** Jian Zhang, Hao Peng, Wen-Fei Li, Yuan Zhang, Li-Zhi Liu, Li Tian, Ai-Hua Lin, Ying Sun, Jun Ma

**Affiliations:** 1 Department of Radiation Oncology, Affiliated Cancer Hospital & Institute of Guangzhou Medical University, Guangzhou, 510000 People’s Republic of China; 2 Department of Radiation Oncology, Sun Yat-sen University Cancer Center, State Key Laboratory of Oncology in Southern China, Collaborative Innovation Center for Cancer Medicine, Guangdong Key Laboratory of Nasopharyngeal Carcinoma Diagnosis and Therapy, Guangzhou, 510060 People’s Republic of China; 3 Imaging Diagnosis and Interventional Center, Sun Yat-sen University Cancer Center, State Key Laboratory of Oncology in Southern China, Collaborative Innovation Center for Cancer Medicine, Guangzhou, People’s Republic of China; 4 Department of Medical Statistics and Epidemiology, School of Public Health, Sun Yat-sen University, Guangzhou, People’s Republic of China; 5 State Key Laboratory of Oncology in South China, Department of Radiation Oncology, Sun Yat-sen University Cancer Center, 651 Dongfeng Road East, Guangzhou, 510060 People’s Republic of China

**Keywords:** Nasopharyngeal carcinoma, Locoregionally advanced, Induction chemotherapy, Epstein-Barr virus DNA, Prognosis

## Abstract

**Background:**

The role of pretreatment Epstein-Barr virus DNA (pre-DNA) for individualized induction chemotherapy (IC) in locoregionally advanced nasopharyngeal carcinoma (LA-NPC) still remains unknown. We aimed to address this clinical issue.

**Methods:**

In total, data on 6218 patient with newly diagnosed LA-NPC receiving concurrent chemoradiotherapy (CCRT) with or without IC were retrospectively reviewed. Receiver operating characteristics (ROC) curve was adopted to calculate the cut-off value of pre-DNA based on disease-free survival (DFS). Propensity score matching (PSM) method was adopted to balance prognostic factors and match patients. Survival outcomes between IC + CCRT and CCRT groups were compared.

**Results:**

Among the original cohort, no survival difference between IC + CCRT and CCRT groups was found. The cut-off value of pre-DNA was 4650 copies/ml (area under curve [AUC], 0.620; sensitivity, 0.6224; specificity, 0.5673). For patients with Pre-DNA ≤ 4650 copies/ml, the IC + CCRT and CCRT groups also achieved comparable survival outcomes (*P* > 0.05 for all rates). However, IC + CCRT was associated with significantly improved 3-year DFS (78.6% vs. 74.8%, *P* = 0.03), overall survival (OS; 91.4% vs. 87.5%, *P* = 0.002) and distant metastasis-free survival (DMFS; 86.0% vs. 82.2%, *P* = 0.036) for patient with pre-DNA > 4650 copies/ml. Multivariate analysis also confirm that IC + CCRT was an independent prognostic factor for DFS (HR, 0.817; 95% CI, 0.683–0.977; *P* = 0.027), OS (HR, 0.675; 95% CI, 0.537–0.848; *P* = 0.001) and DMFS (HR, 0.782; 95% CI, 0.626–0.976; *P* = 0.03).

**Conclusions:**

Pre-DNA may be a feasible and powerful consideration for individualized IC apart from other baseline clinical characteristics in LA-NPC.

**Electronic supplementary material:**

The online version of this article (10.1186/s12885-018-5177-9) contains supplementary material, which is available to authorized users.

## Background

Nasopharyngeal carcinoma (NPC) is a malignancy arising from nasopharynx epithelia, and epidemic in Southeast and Eastern Asia. The highest incidence occurred in Southeast China and is approximately 20–50 per 100,000 people [1, 2]. Radiation therapy (RT) is the primary and only curative treatment for non-disseminated disease as a result of its complicate anatomy location and sensitivity to irradiation. Concurrent chemoradiotherapy (CCRT) is now the main treatment for locoregionally advanced NPC (LA-NPC) [3, 4]. However, prognosis of LA-NPC after radical radiotherapy still remains poor [5] and distant metastasis is the main failure pattern [6]. To further decrease risks of distant metastasis and improve clinical outcomes, induction chemotherapy (IC) additional to CCRT has been proven a feasible and effective strategy [7–9]. Notably, there is increasing data showing that IC additional to CCRT could not bring therapeutic gain to patients with T3–4 N0–1 disease [10, 11], indicating that some patients with low risk did not need IC. However, current risk stratification and treatment delivery mainly refer to TNM staging system which may be insufficient to identify the low-risk patients [12]. Therefore, it is urgently needed to identify powerful factors to help risk stratification and treatment strategy selection.

Plasma Epstein-Barr virus (EBV) DNA has been proven an important factor in risk stratification and prognosis prediction in NPC [13–15]. Moreover, plasma EBV DNA could also play an important role in decision making. For example, post-treatment EBV DNA could act as an indicator for individualized adjuvant chemotherapy [16]. Recently, Guo et al. [17] and Peng et al. [18] found that pre-treatment Epstein-Barr virus (pre-DNA) could guide the selection of IC in LA-NPC. However, the sample size in these two studies was small. Moreover, the treatment modality was also not uniform since many patients did not received concurrent chemotherapy, which would subject the study to treatment-related bias. Therefore, it is necessary to further address this question and provide robust evidence.

Based on this premise, we conducted this retrospective study using a big-data, intelligence database platform to identify and evaluate the value of pre-DNA for risk stratification and treatment selection in LA-NPC.

## Methods

### Patient selection

In this study, we reviewed and identified patients with newly diagnosed stage I-IVA NPC who were treated between November 2009 and February 2015 using the big-data, intelligence platform at Sun Yat-sen University Cancer Center [19]. Patients meeting the following criteria were included for this study: (1) newly diagnosed stage III-IVA NPC; (2) data on pre-DNA was available; (3) receiving intensity-modulated radiotherapy (IMRT); (4) age 18 years or older; (5) receiving CCRT with or without IC; (6) the cycles of IC should be ≥2. Finally, 6218 patients were recruited for the current study. This study was approved by the Research Ethics Committee of our center. Informed consent was obtained from all the patients. Study data was deposited at the Research Data Deposit platform (http://www.researchdata.org.cn/, RDDA2018000545).

### Clinical staging

Before treatment, patients received physical examination first. Then imaging methods were performed including magnetic resonance imaging (MRI) of the neck and nasopharynx, whole-body bone scan, abdominal sonography or computed tomograph, chest radiography or tomograph. Positron emission tomography (PET)-CT would also be recommended if clinically indicated. Imaging data were reviewed by two radiologists (L-ZL and LT) independently to stage all patients based on the 8th edition of the International Union against Cancer/American Joint Committee on Cancer (UICC/AJCC) staging system manual.

### Real-time quantitative EBV DNA PCR

Pre-DNA concentration was detected using real-time quantitative polymerase chain reaction (RT-PCR) which was described previously [20]. The RT-PCR system was developed and targeted the *BamH*I-W region of the EBV genome using primers 5’-GCCAGAGGTAAGTGGACTTT-3′ and 5’-TACCACCTCCTCTTCTTGCT-3′. The dual fluorescence-labeled oligomer 5′-(FAM) CACACCCAGGCACACACTACACAT (TAMRA)-3′ served as a probe. Sequence data for the EBV genome were obtained from the GeneBank sequence database.

### Clinical treatment

All patients underwent radical IMRT. The prescribed radiation doses were 66 Gy or greater to the primary tumor and 60–70 Gy to the involved neck area. All potential sites of local infiltration and bilateral cervical lymphatics were irradiated to 50 Gy or greater. All patients were treated with 30–35 fractions with five daily fractions per week for 6–7 weeks.

Since our study is retrospective and patients were treated before 2016 when the role of IC has not been well established. Therefore, the selection of IC and corresponding regimens mainly depended on clinicians’ experience and decisions because there was no consensus in our center. IC regimens consist of platinum-based agents including 5-fluorouracil with cisplatin (PF), docetaxel with cisplatin (TP) and triple of docetaxel with 5-fluorouracil and cisplatin (TPF). Concurrent chemotherapy consisted of weekly (30–40 mg/m^2^ d1) or tri-weekly (80–100 mg/m^2^ d1) cisplatin.

### Follow-up strategy

Patients were followed by imaging methods every 3 months during first 2 years, 6 months during 3-5th year and annually thereafter. Follow-up duration was measured from first day of pathological diagnosis to last visit or death. The first endpoint is disease-free survival (DFS, defined as the time to first event or death from any cause). Other endpoints include overall survival (OS, time to death from any cause), distant metastasis-free survival (DMFS, time to first distant failure) and locoregional relapse-free survival (LRRFS, time to first local or regional recurrence or both).

### Statistical method

Propensity score matching (PSM) using logistic regression were adopted to balance factors and match patients. The Chi-square test or Fisher’s exact test were used to compare categorical variables and non-parametric test for continuous variables. Receiver operation characteristic (ROC) curve was applied to calculate the cut-off value of pre-DNA for DFS. Life-table estimation was performed using the Kaplan-Meier method and survival difference was compared by log-rank test. The multivariate Cox proportional hazards model was used to estimate hazard ratios (HRs) and 95% confidence intervals (CIs) with following factors; gender, age, smoking, drinking, family history of cancer, lactate dehydrogenase (LDH), T category, N category, overall stage, and treatment arms (IC + CCRT vs. CCRT). All tests were two-sided; *P* <  0.05 was considered significant. Statistical Package 12 (StataCorp LP, College Station, TX, USA) was used for all analyses.

## Results

### Baseline characteristics

In total, 6218 patients with LA-NPC were included in this study and baseline characteristics were summarized in Additional file 1: Table S1. The whole cohort carried a male-to-female ratio of 2.86, and the median age was 45 (range, 18–79) yeas-old. The median follow-up duration was 43.0 (range, 0.3–103.6) months. Overall, 3510 (56.4%) patients received IC + CCRT and 2708 (43.6%) received CCRT alone. Specifically, 1460 (41.6%), 977 (27.8%) and 1073 (30.6%) patients received induction TPF, PF and TP regimens, respectively. Obviously, the IC + CCRT group had a higher percentage of T4, N3 and stage IV disease (*P* <  0.001). Besides, host and tumor-related factors were also not balanced between these two groups (*P* <  0.05).

### Survival analysis within whole cohort

**Fig. 1:**
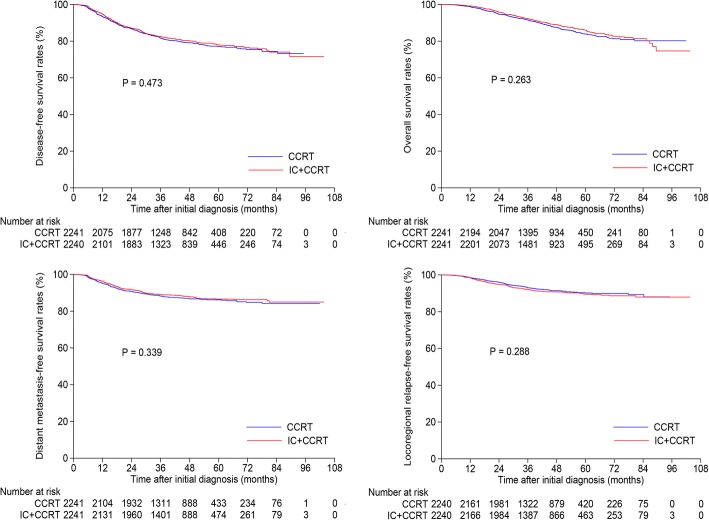
Kaplan-Meier disease-free survival, overall survival, distant metastasis-free survival and locoregional relapse-free survival curves for the selected 2241 pairs with stage III-IVA nasopharyngeal carcinoma receiving concurrent chemoradiotherapy with or without induction chemotherapy

First, we compared the survival outcomes of IC + CCRT with that of CCRT among the original cohort. In total, 2241 pairs were selected by PSM from the 6218 patients (Table 1), and factors were well balanced between the two groups. The 3-year DFS (82.5% vs. 81.7%, *P* = 0.473), OS (92.3% vs. 91.6%, *P* = 0.263), DMFS (89.1% vs. 88.2%, *P* = 0.339) and LRRFS (92.0% vs. 93.1%, *P* = 0.288) (Fig. 1) rates were comparable between IC + CCRT and CCRT groups. When multivariate analysis performed, results were consisted with that of univariate analysis and treatment arm (IC + CCRT vs. CCRT) was not an independent prognostic factor for DFS, OS, DMFS and LRRFS (Table 2).

**Table 1:** Baseline characteristics of selected 2241 pairs with locoregionally advanced nasopharyngeal carcinoma

Characteristics	CCRT (*n* = 2241)	IC + CCRT (n = 2241)	*P* value
	No. (%)	No. (%)	
Gender			0.838^a^
Male	585 (26.1)	579 (25.8)	
Female	1656 (73.9)	1662 (74.2)	
Age (years)			0.791^b^
Median (range)	44 (18–77)	45 (18–76)	
Smoking			0.828^a^
Yes	819 (36.5)	812 (36.2)	
No	1422 (63.5)	1429 (63.8)	
Drinking			0.703^a^
Yes	321 (14.3)	330 (14.7)	
No	1920 (85.7)	1911 (85.3)	
Family History of cancer			0.606^a^
Yes	572 (25.5)	557 (24.9)	
No	1669 (74.5)	1684 (75.1)	
T category ^c^			0.208^a^
T1	107 (4.8)	136 (6.0)	
T2	179 (8.0)	176 (7.9)	
T3	1461 (65.2)	1416 (63.2)	
T4	494 (22.0)	513 (22.9)	
N category ^c^			0.03^a^
N0	186 (8.3)	188 (8.4)	
N1	1151 (51.4)	1138 (50.8)	
N2	635 (28.3)	699 (31.2)	
N3	269 (12.0)	216 (9.6)	
Overall stage ^c^			0.501^a^
III	1516 (67.6)	1537 (68.6)	
IVA-B	725 (32.4)	704 (31.4)	
LDH (U/L)			0.204^b^
Median (range)	174 (67–1009)	176 (39–753)	
EBV-DNA (copies/ml)			0.141^b^
Median (range)	6345 (0–13,100,000)	4610 (0–9,080,000)	

*Abbreviations*: *NPC* nasopharyngeal carcinoma, *CCRT* concurrent chemoradiotherapy, *IC* induction chemotherapy, *LDH* lactate dehydrogenase, *EBV-DNA* Epstein-Barr virus DNA

^a^*P* values were calculated by Chi-square test

^b^*P* values were calculated by t test

^c^According to the 8th edition of UICC/AJCC staging system

**Table 2:** Results of multivariate analysis for the selected 2241 pairs

Endpoints	Variable	HR (95% CI)	*P* value ^a^
DFS	Gender, female vs. male	0.799 (0.681–0.937)	0.006
	Age, > 44 vs. ≤ 44y	1.191 (1.041–1.362)	0.011
	LDH; > 245 vs. ≤ 245 U/L	1.596 (1.298–1.962)	< 0.001
	T category; T3–4 vs. T1–2	1.283 (1.043–1.578)	0.018
	N category, N2–3 vs. N0–1	1.742 (1.506–2.015)	< 0.001
	Overall stage, IVA vs. III	1.867 (1.632–2.136)	< 0.001
	Treatment, IC + CCRT vs. CCRT	0.955 (0.837–1.090)	0.494
OS	Gender; female vs. male	0.700 (0.562–0.871)	0.001
	Age, > 44 vs. ≤ 44y	1.479 (1.238–1.768)	< 0.001
	LDH; > 245 vs. ≤ 245 U/L	1.734 (1.343–2.238)	< 0.001
	N category, N2–3 vs. N0–1	1.922 (1.589–2.323)	< 0.001
	Overall stage, IVA vs. III	2.140 (1.795–2.550)	< 0.001
	Treatment, IC + CCRT vs. CCRT	0.894 (0.752–1.063)	0.207
DMFS	Gender; female vs. male	0.758 (0.616–0.932)	0.009
	LDH; > 245 vs. ≤ 245 U/L	1.938 (1.519–2.473)	< 0.001
	N category, N2–3 vs. N0–1	1.959 (1.649–2.327)	< 0.001
	Overall stage, IVA vs. III	1.989 (1.678–2.357)	< 0.001
	Treatment, IC + CCRT vs. CCRT	0.926 (0.782–1.096)	0.369
LRRFS	Smoking, yes vs. no	1.263 (1.024–1.557)	0.029
	Age, > 44 vs. ≤ 44y	1.345 (1.088–1.662)	0.006
	T category, T3–4 vs. T1–2	1.494 (1.062–2.103)	0.021
	N category; N3 vs. N2	1.624 (1.297–2.033)	< 0.001
	Overall stage, IVA vs. III	1.654 (1.341–2.039)	< 0.001
	Treatment, IC + CCRT vs. CCRT	1.114 (0.906–1.369)	0.306

*Abbreviations*: *DFS* disease-free survival, *OS* overall survival, *DMFS* distant metastasis-free survival, *LRRFS* locoregional relapse-free survival, *HR* hazard ratio, *CI* confidence interval, *IC* induction chemotherapy, *CCRT* concurrent chemoradiotherapy, *LDH* lactate dehydrogenase

^a^*P*-values were calculated using an adjusted Cox proportional hazards model with backward elimination and the following variables were included: gender (female vs. male), age (> 44y vs. ≤ 44y), smoking (yes vs. no), drinking (yes vs. no), family history of cancer (yes vs. no), LDH (> 245 vs. ≤ 245 U/L), T category (T3–4 vs. T1–2), N category (N2–3 vs. N0–1), overall stage (IVA vs. III) and treatment (IC + CCRT vs. CCRT)

### Cut-off value of pre-DNA

**Fig. 2:**
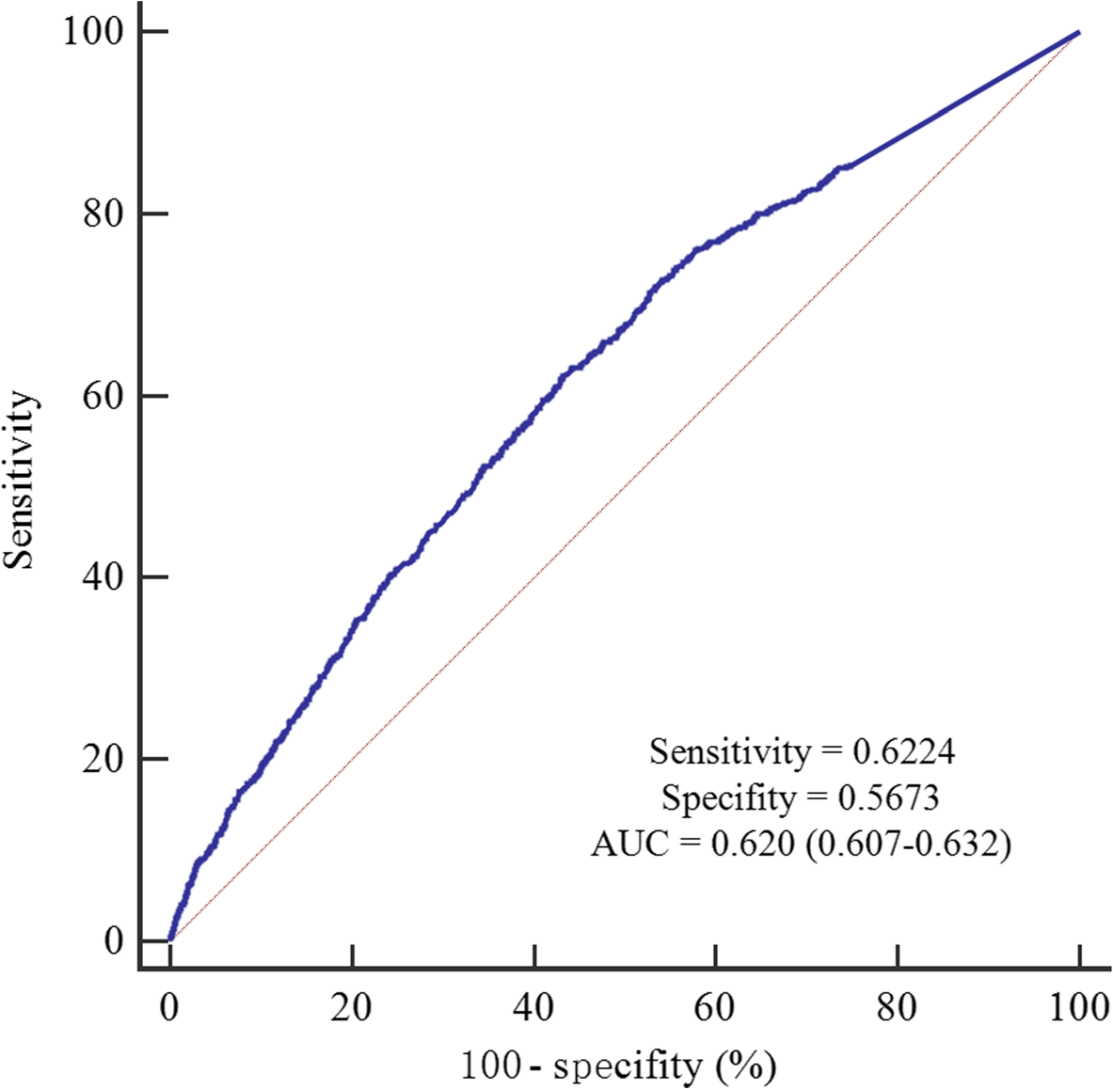
Receiver operation characteristic (ROC) curve analysis for identifying the cut-off value of pre-treatment Epstein-Barr virus DNA

The median pre-DNA concentration for the 6218 patients was 3740 (range, 0–68,700,000) copies/ml. Based on ROC analysis, the cut-off value of pre-DNA is 4650 copies/ml (sensitivity = 0.6224, specificity = 0.5673, area under curve [AUC] = 0.620) for DFS (Fig. 2), 4315 (sensitivity: 0.667; specificity: 0.545; AUC = 0.634) for OS, 4315 (sensitivity: 0.674; specificity: 0.547; AUC = 0.649) for DMFS and 2055 (sensitivity: 0.680; specificity: 0.446; AUC = 0.568). Then, 4650 copies/ml was used as the threshold. We further evaluated whether this cut-off value could subdivide patients into different risk groups. Undoubtedly, patients with pre-DNA > 4650 copies/ml achieved significantly poorer survival compared with those with pre-DNA ≤ 4650 copies/ml in both stage III (Additional file 2: Figure S1) and stage IV (Additional file 3: Figure S2) subgroups. Therefore, this cut-off value is valid, and patients with pre-DNA ≤ 4650 copies/ml were classified as low-DNA group and those with pre-DNA > 4650 copies/ml as high-DNA group.

### Survival outcomes within low-DNA group

**Fig. 3:**
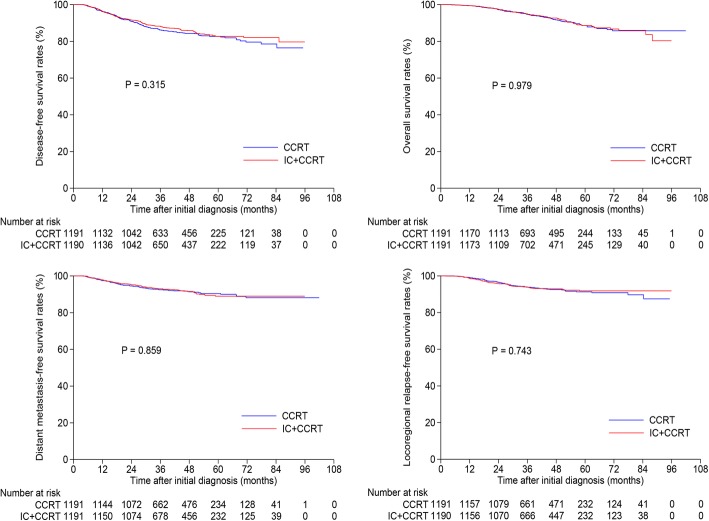
Kaplan-Meier disease-free survival, overall survival, distant metastasis-free survival and locoregional relapse-free survival curves for the selected 1191 pairs with pre-DNA ≤ 4650 copies/ml receiving concurrent chemoradiotherapy with or without induction chemotherapy. Pre-DNA, pre-treatment Epstein-Barr virus DNA

We further evaluated the survival difference between the IC + CCRT and CCRT groups among patients with low-DNA. In total, 3292 patients had a pre-DNA ≤ 4650 copies/ml (Additional file 4: Table S2). After matching, 1191 pairs were selected and baseline information was presented in Additional file 5: Table S3. The 3-year DFS, OS, DMFS and LRRFS rates for IC + CCRT vs. CCRT were 88.2% vs. 86.2% (*P* = 0.315), 95.0% vs. 94.7% (*P* = 0.979), 93.0% vs. 92.5% (*P* = 0.859) and 93.8% vs. 93.9% (*P* = 0.743; Fig. 3), respectively. Multivariate analysis also found that there was no significantly survival difference between IC + CCRT and CCRT groups (*P* > 0.05 for all rates, Additional file 6: Table S4). Therefore, IC + CCRT and CCRT achieved similar outcomes in low-DNA group.

### Survival outcomes within high-DNA group

**Fig. 4:**
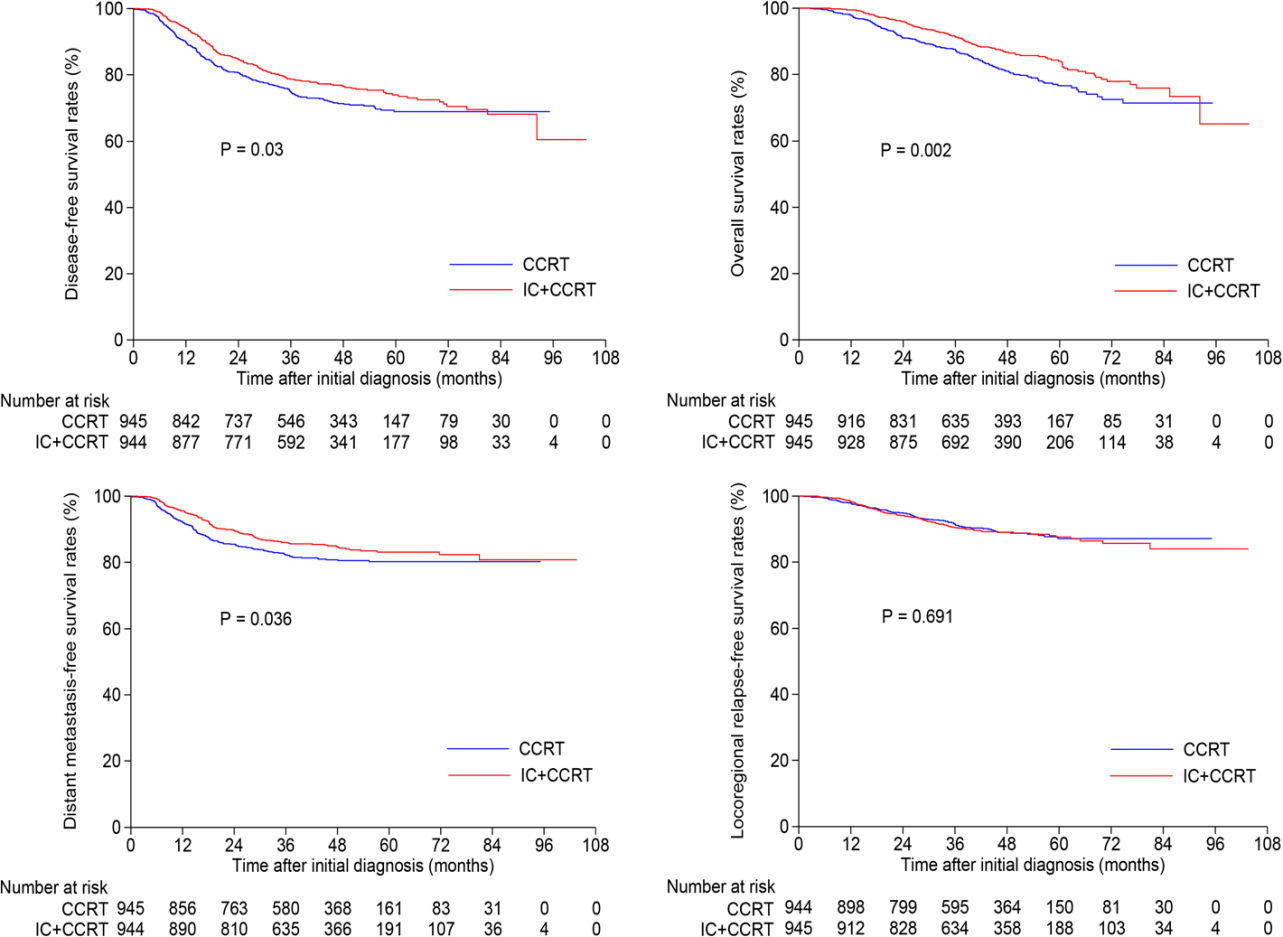
Kaplan-Meier disease-free survival, overall survival, distant metastasis-free survival and locoregional relapse-free survival curves for the selected 945 pairs with pre-DNA > 4650 copies/ml receiving concurrent chemoradiotherapy with or without induction chemotherapy. Pre-DNA, pre-treatment Epstein-Barr virus DNA

Among the 2926 patients with pre-DNA > 4650 copies/ml (Additional file 7: Table S5), 945 pairs were selected by PSM and baseline characteristics were presented in Additional file 8: Table S6. The 3-year DFS, OS, DMFS and LRRFS rates for IC + CCRT vs. CCRT were 78.6% vs. 74.8% (*P* = 0.03), 91.4% vs. 87.5% (*P* = 0.002), 86.0% vs. 82.2% vs. (*P* = 0.036) and 90.4% vs. 91.4% (*P* = 0.691; Fig. 4), respectively. When entered into multivariate analysis, treatment (IC + CCRT vs. CCRT) was identified as an independent prognostic factor for DFS (HR, 0.817; 95% CI, 0.683–0.977; *P* = 0.027), OS (HR, 0.675; 95% CI, 0.537–0.848; *P* = 0.001) and DMFS (HR, 0.782; 95% CI, 0.626–0.976; *P* = 0.03; Table 3). Thus, IC + CCRT was superior to CCRT among patients with high-DNA.

**Table 3:** Results of multivariate analysis for the selected 945 pairs with pre-treatment Epstein-Barr virus DNA > 4650 copies/ml

Endpoints	Variable	HR (95% CI)	*P* value ^a^
DFS	Gender, female vs. male	0.694 (0.559–0.862)	0.001
	Family history of cancer, yes vs. no	1.273 (1.042–1.555)	0.018
	LDH; > 245 vs. ≤ 245 U/L	1.345 (1.050–1.723)	0.019
	N category, N2–3 vs. N0–1	1.693 (1.405–2.039)	< 0.001
	Overall stage, IVA vs. III	1.349 (1.125–1.617)	0.001
	Treatment, IC + CCRT vs. CCRT	0.817 (0.683–0.977)	0.027
OS	Gender, female vs. male	0.630 (0.472–0.841)	0.002
	Age, > 44 vs. ≤ 44y	1.499 (1.188–1.892)	0.001
	LDH; > 245 vs. ≤ 245 U/L	1.510 (1.118–2.039)	0.007
	N category, N2–3 vs. N0–1	1.820 (1.437–2.305)	< 0.001
	Overall stage, IVA vs. III	1.396 (1.110–1.757)	0.004
	Treatment, IC + CCRT vs. CCRT	0.675 (0.537–0.848)	0.001
DMFS	Gender, female vs. male	0.674 (0.513–0.886)	0.005
	LDH; > 245 vs. ≤ 245 U/L	1.658 (1.245–2.209)	0.001
	N category, N2–3 vs. N0–1	1.874 (1.481–2.372)	< 0.001
	Overall stage, IVA vs. III	1.377 (1.100–1.725)	0.005
	Treatment, IC + CCRT vs. CCRT	0.782 (0.626–0.976)	0.03
LRRFS	Gender, female vs. male	0.598 (0.416–0.859)	0.005
	Family history of cancer, yes vs. no	1.533 (1.128–2.083)	0.006
	N category; N3 vs. N2	1.712 (1.274–2.301)	< 0.001
	Treatment, IC + CCRT vs. CCRT	1.063 (0.798–1.415)	0.677

*Abbreviations*: *DFS* disease-free survival, *OS* overall survival, *DMFS* distant metastasis-free survival, *LRRFS* locoregional relapse-free survival, *HR* hazard ratio, *CI* confidence interval, *IC* induction chemotherapy, *CCRT* concurrent chemoradiotherapy, *LDH* lactate dehydrogenase

^a^*P*-values were calculated using an adjusted Cox proportional hazards model with backward elimination and the following variables were included: gender (female vs. male), age (> 44y vs. ≤ 44y), smoking (yes vs. no), drinking (yes vs. no), family history of cancer (yes vs. no), LDH (> 245 vs. ≤ 245 U/L), T category (T3–4 vs. T1–2), N category (N2–3 vs. N0–1), overall stage (IVA vs. III) and treatment (IC + CCRT vs. CCRT)

## Discussion

Our current study presented that patients with LA-NPC and low pre-DNA (≤ 4650 copies/ml) could not benefit from additional IC to CCRT while patients with high pre-DNA (> 4650 copies/ml) could, indicating that pre-DNA could act as an effective and powerful indicator for the delivery of IC in LA-NPC. Notably, to avoid extended follow-up and identify a cut-off value for earlier individualized treatment, we therefore calculated the cut-off value of pre-DNA based on DFS because it was a feasible surrogate endpoint for OS [21, 22]. To the best of our knowledge, this is the largest cohort study in evaluating the role of pre-DNA for treatment strategies selection.

In the era of IMRT, distant metastasis has emerged as the predominant treatment failure pattern, especially for advanced disease [23, 24]. Additional cycles of chemotherapy to CCRT are needed to reduce distant metastasis and further improve survival. Adjuvant chemotherapy (AC) was firstly considered as it was proven effective by Intergroup 0099 study [3]. However, subsequent studies found that AC additional to CCRT may be useless [25, 26]. Furthermore, the severe toxicities of AC constrain its wide usage. Given these concerns, other chemotherapy strategies with better efficacy and compliance should be identified. IC, delivered before radiotherapy, has caught a lot of attention for its better compliance and early eradication of subclinical micro-metastasis. However, results from previous clinical trials comparing IC + CCRT with CCRT were controversial as the three achieved positive outcomes [8, 27, 28] while the study by Tan et al. [29] achieved negative results, indicating that not all the patients with LA-NPC could benefit from IC. Moreover, retrospective evidence showed that IC could not produce therapeutic gain for patients with T3–4 N0–1 [10, 11], further revealing that great heterogeneity exists in patients with LA-NPC. Therefore, effective factors should be identified to subdivide patients with different risk groups and then deliver IC. Our study proved pre-DNA could act as that factor.

It is well known that NPC is an EBV-driven malignancy [30, 31]. Our study selected pre-DNA as the only indicator for IC because the prognostic role of EBV DNA in NPC has been proven by numerous studies [13, 32–34]. Possibly, EBV DNA has been the strongest and most widely used factor in NPC so far. Although many other prognostic factors like LDH [35, 36] and tumor volume [37] have also been proven effective, they were not been widely proven and evidence supporting them were not too much. We therefore only selected pre-DNA as the indicator. Notably, the cut-off value of pre-DNA in our study was different from that used in other studies [17, 38] because we calculate it based on our data. The cut-off value of 4000 copies/ml used in the two studies [17, 38] came from other literatures and was not calculated based on their own data. Thus, our results may be more credible and reflect intrinsic relationship of pre-DNA and IC. However, it should be pointed out that common calibrators and PCR master mix should be warranted to reduce variability in plasma EBV DNA numbers [39] before our cut-off value could be applied widely and uniformly.

Among the original cohort without stratification by pre-DNA, no significant survival difference between IC + CCRT and CCRT groups was observed. One possible explanation may be that some patients with low-risk could not benefit from IC and they counteract the benefit for high-risk patients, resulting in non-significant difference. When stratified analysis according to pre-DNA was performed, a different scenario happened. Among patients with low-DNA, IC + CCRT and CCRT achieved comparable survival outcomes; while for those with high-DNA, IC + CCRT group achieved significantly better DFS, OS and DMFS than CCRT group. These results were consistent with previous studies [17, 18, 38]. Undoubtedly, patients with high-DNA had higher tumor burden and risk of distant metastasis, therefore could benefit from IC. Our findings together with previous studies [17, 18, 38] further supported that pre-DNA could act as a strong and reliable indicator for IC.

Compared with previous studies [17, 18, 38], our study mainly had two advantages. First, all the patients received standard treatment (i.e., CCRT-based regimen was delivered to all patients), thus reducing treatment-related bias. Second, the sample size is large, therefore achieving more powerfully statistical results. By applying PSM and multivariate analysis, we addressed the potential limitations of divergent confounders, treatment heterogeneity and selection bias associated with retrospective analysis of observational data [40]. The limitations in this study should also be acknowledged. First, our study is retrospective, meaning potential bias may exist. Moreover, the follow-up duration may not be long enough which would produce few events and prevent data from reach statistically significant. Therefore, a longer follow-up length is necessary to further evaluate the role of pre-DNA for IC. Finally, completion of concurrent chemotherapy between IC + CCRT and CCRT groups was not addressed in our study. As shown by previous study, IC could affect the completion of tri-weekly cisplatin regimen (100 mg/m^2^) [28]. However, the concurrent regimen used in our study was different from that. Therefore, this issue should be addressed in future study.

## Conclusion

In summary, our study revealed that patients with high pre-DNA could benefit from additional IC to CCRT while those with low pre-DNA could not in LA-NPC in the era of IMRT, indicating pre-DNA may be a feasible and powerful consideration for individualized IC apart from other baseline clinical characteristics. Future randomized clinical trials are warranted to validate our findings.

## Additional files

Additional file 1: **Table S1.**Baseline characteristics of 6218 patients with locoregionally advanced nasopharyngeal carcinoma. (DOCX 16 kb)

Additional file 2: **Figure S1.**Kaplan-Meier disease-free survival, overall survival, distant metastasis-free survival and locoregional relapse-free survival curves stratified as pre-DNA ≤ 4650 or > 4650 copies/ml for patients with stage III nasopharyngeal carcinoma. Pre-DNA, pre-treatment Epstein-Barr virus DNA. (TIF 417 kb)

Additional file 3: **Figure S2.**Kaplan-Meier disease-free survival, overall survival, distant metastasis-free survival and locoregional relapse-free survival curves stratified as pre-DNA ≤ 4650 or > 4650 copies/ml for patients with stage IVA nasopharyngeal carcinoma. Pre-DNA, pre-treatment Epstein-Barr virus DNA. (TIF 426 kb)

Additional file 4: **Table S2.**Baseline characteristics of 3292 patients with pre-treatment Epstein-Barr virus DNA ≤ 4650 copies/ml. (DOCX 16 kb)

Additional file 5: **Table S3.**Baseline characteristics of selected 1191 pairs with pre-treatment Epstein-Barr virus DNA ≤ 4650 copies/ml. (DOCX 16 kb)

Additional file 6: **Table S4.**Results of multivariate analysis for the selected 1191 pairs with pre-treatment Epstein-Barr virus DNA ≤ 4650 copies/ml. (DOCX 15 kb)

Additional file 7: **Table S5.**Baseline characteristics of 2926 patients with pre-treatment Epstein-Barr virus DNA > 4650 copies/ml. (DOCX 16 kb)

Additional file 8: **Table S6.**Baseline characteristics of selected 945 pairs with pre-treatment Epstein-Barr virus DNA > 4650 copies/ml. (DOCX 16 kb)

## Abbreviations

CCRT: Concurrent chemoradiotherapy
CI: Confidence intervals
DFS: Disease-free survival
DMFS: Distant metastasis-free survival
EBV: Epstein-Barr virus
HR: Hazard ratios
IC: Induction chemotherapy
IMRT: Intensity-modulated radiotherapy
LA-NPC: Locoregionally advanced nasopharyngeal carcinoma
LDH: Lactate dehydrogenase
LRRFS: Locoregional relapse-free survival
MRI: Magnetic resonance imaging
NPC: Asopharyngeal carcinoma
OS: Overall survival
PCR: Polymerase chain reaction
PET: Positron emission tomography
PF: 5-fluorouracil with cisplatin
Pre-DNA: Pre-treatment Epstein-Barr virus
ROC: Receiver operation characteristic
RT: Radiation therapy
TP: Docetaxel with cisplatin
TPF: Docetaxel with 5-fluorouracil and cisplatin
UICC/AJCC: International Union against Cancer/American Joint Committee on Cancer
WHO: World Health Organization
