# Supplementary material for: Individualized induction chemotherapy by pre-treatment plasma Epstein-Barr viral DNA in advanced nasopharyngeal carcinoma
Source: BMC Cancer. 2018 Dec 19;18:1276. doi: 10.1186/s12885-018-5177-9 (PMC6299978; doi:10.1186/s12885-018-5177-9)
Supplement: Supplementary file 1 — Table S1.Baseline characteristics of 6218 patients with locoregionally advanced nasopharyngeal carcinoma. (DOCX 16 kb) [file 12885_2018_5177_MOESM1_ESM.docx]

**Table S1**. Baseline characteristics of 6218 patients with locoregionally advanced nasopharyngeal carcinoma.

| Characteristics | CCRT (n=2708) | | IC+CCRT (n=3510) | | *P* value |
| --- | --- | --- | --- | --- | --- |
|  | No. (%) | | No. (%) | |  |
| Gender |  | |  | | 0.002^a^ |
| Female | 754 (27.8) | | 857 (24.4) | |  |
| Male | 1954 (72.2) | | 2653 (75.6) | |  |
| Age (years) |  | |  | | 0.179^b^ |
| Median (range) | 45 (18-79) | | 44 (18-77) | |  |
| Smoking |  | |  | | 0.003^a^ |
| Yes | 957 (35.3) | | 1369 (39.0) | |  |
| No | 1751 (64.7) | | 2141 (61.0) | |  |
| Drinking |  | |  | | 0.433^a^ |
| Yes | 412 (15.2) | | 509 (14.5) | |  |
| No | 2296 (84.8) | | 3001 (85.5) | |  |
| Family History of cancer | |  | | 0.420^a^ | |
| Yes | 690 (25.5) | | 863 (24.6) | |  |
| No | 2018 (74.5) | | 2647 (75.4) | |  |
| T category ^c^ |  | |  | | < 0.001^a^ |
| T1 | 140 (5.2) | | 179 (5.1) | |  |
| T2 | 189 (7.0) | | 272 (7.7) | |  |
| T3 | 1885 (69.6) | | 1844 (52.5) | |  |
| T4 | 494 (18.2) | | 1215 (34.7) | |  |
| N category ^c^ |  | |  | | < 0.001^a^ |
| N0 | 354 (13.1) | | 213 (6.1) | |  |
| N1 | 1400 (51.7) | | 1469 (41.9) | |  |
| N2 | 684 (25.3) | | 1079 (30.7) | |  |
| N3 | 270 (9.9) | | 748 (21.3) | |  |
| Overall stage ^c^ |  | |  | | < 0.001^a^ |
| III | 1982 (73.2) | | 1706 (48.6) | |  |
| IVA-B | 726 (26.8) | | 1804 (51.4) | |  |
| LDH (U/L) |  | |  | | < 0.001^b^ |
| Median (range) | 175 (67-1009) | | 179 (39-774) | |  |
| EBV-DNA (copies/ml) | | |  | | < 0.001^b^ |
| Median (range) | 6345 (0-68700000) | | 1500 (0-13100000) | |  |

Abbreviations: NPC = nasopharyngeal carcinoma; CCRT = concurrent chemoradiotherapy; IC = induction chemotherapy; LDH = lactate dehydrogenase; EBV-DNA = Epstein-Barr virus DNA.

^a^ *P* values were calculated by Chi-square test.

^b^ *P* values were calculated by t test.

^c^ According to the 8th edition of UICC/AJCC staging system.
