# Supplementary figures and images for: Individualized induction chemotherapy by pre-treatment plasma Epstein-Barr viral DNA in advanced nasopharyngeal carcinoma
Source: BMC Cancer. 2018 Dec 19;18:1276. doi: 10.1186/s12885-018-5177-9 (PMC6299978; doi:10.1186/s12885-018-5177-9)

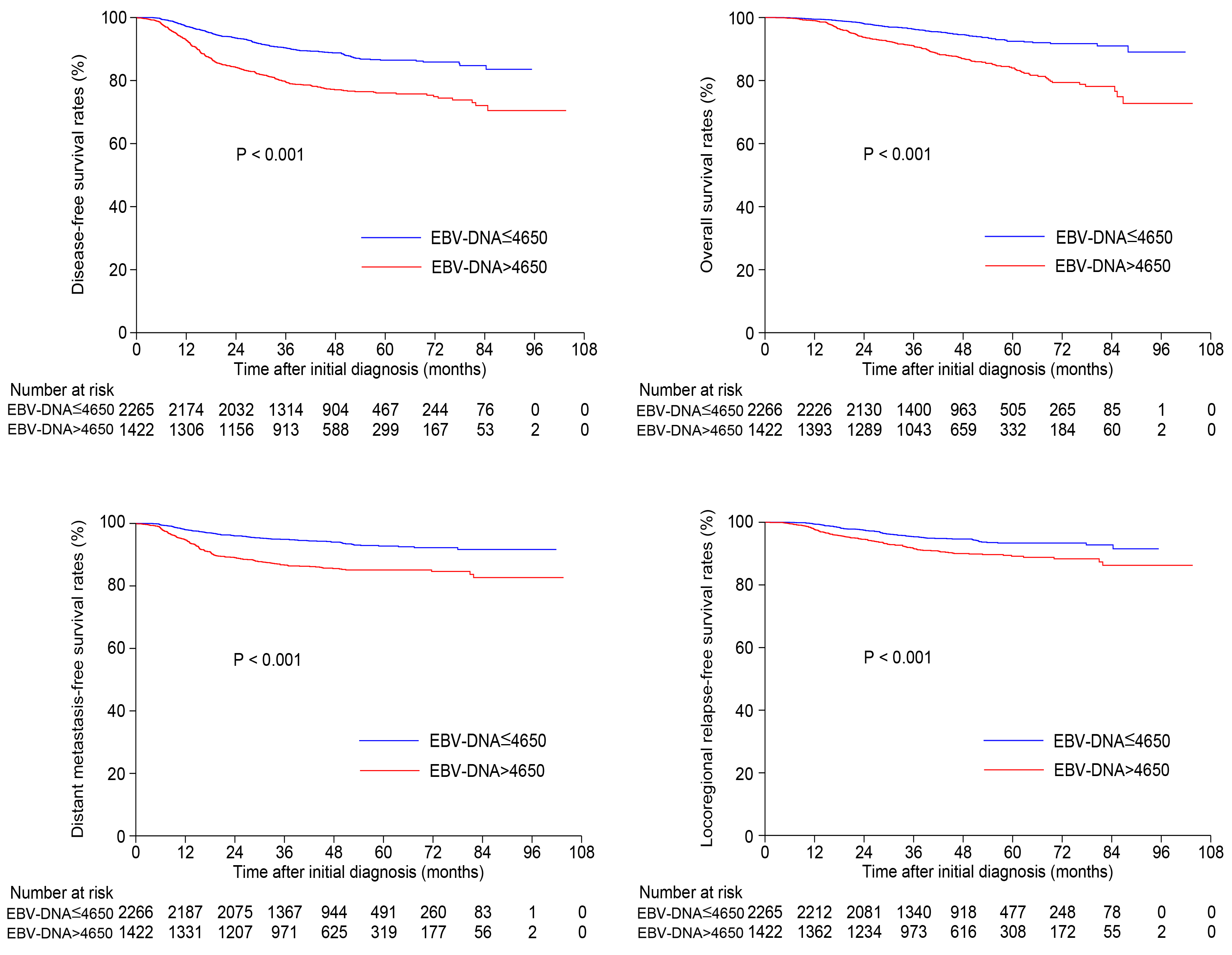

Supplement: Supplementary file 2 — Figure S1.Kaplan-Meier disease-free survival, overall survival, distant metastasis-free survival and locoregional relapse-free survival curves stratified as pre-DNA ≤ 4650 or > 4650 copies/ml for patients with stage III nasopharyngeal carcinoma. Pre-DNA, pre-treatment Epstein-Barr virus DNA. (TIF 417 kb) [file 12885_2018_5177_MOESM2_ESM.tif]

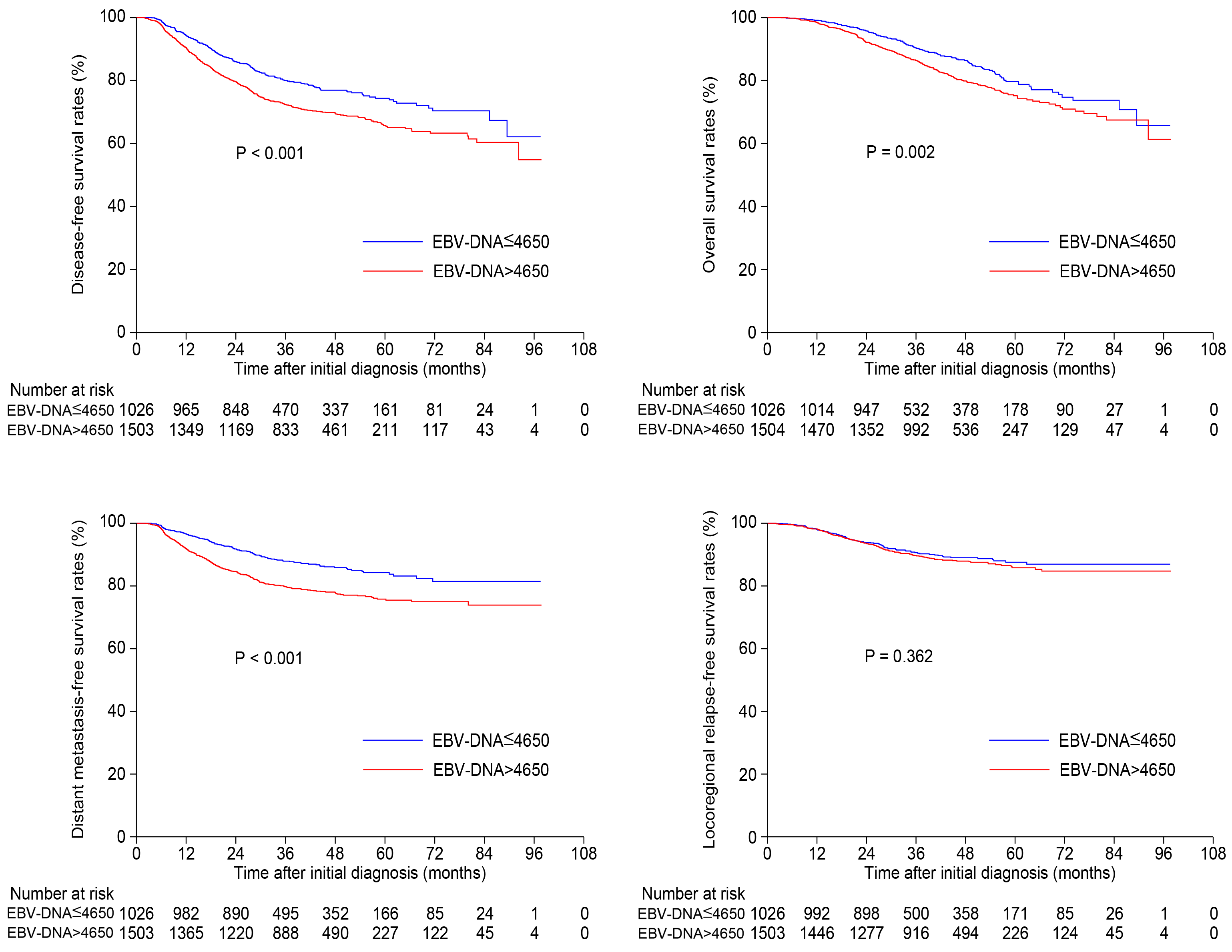

Supplement: Supplementary file 3 — Figure S2.Kaplan-Meier disease-free survival, overall survival, distant metastasis-free survival and locoregional relapse-free survival curves stratified as pre-DNA ≤ 4650 or > 4650 copies/ml for patients with stage IVA nasopharyngeal carcinoma. Pre-DNA, pre-treatment Epstein-Barr virus DNA. (TIF 426 kb) [file 12885_2018_5177_MOESM3_ESM.tif]
