# Supplementary material for: Individualized induction chemotherapy by pre-treatment plasma Epstein-Barr viral DNA in advanced nasopharyngeal carcinoma
Source: BMC Cancer. 2018 Dec 19;18:1276. doi: 10.1186/s12885-018-5177-9 (PMC6299978; doi:10.1186/s12885-018-5177-9)
Supplement: Supplementary file 4 — Table S2.Baseline characteristics of 3292 patients with pre-treatment Epstein-Barr virus DNA ≤ 4650 copies/ml. (DOCX 16 kb) [file 12885_2018_5177_MOESM4_ESM.docx]

**Table S2**. Baseline characteristics of 3292 patients with pre-treatment Epstein-Barr virus DNA ≤ 4650 copies/ml.

| Characteristics | CCRT (n=1709) | | IC+CCRT (n=1583) | | *P* value |
| --- | --- | --- | --- | --- | --- |
|  | No. (%) | | No. (%) | |  |
| Gender |  | |  | | 0.091^a^ |
| Female | 473 (27.7) | | 397 (25.1) | |  |
| Male | 1236 (72.3) | | 1186 (74.9) | |  |
| Age (years) |  | |  | | 0.633^b^ |
| Median (range) | 45 (18-79) | | 44 (18-77) | |  |
| Smoking |  | |  | | 0.323^a^ |
| Yes | 598 (35.0) | | 580 (36.6) | |  |
| No | 1111 (65.0) | | 1003 (63.4) | |  |
| Drinking |  | |  | | 0.811^a^ |
| Yes | 249 (14.6) | | 226 (14.3) | |  |
| No | 1460 (85.4) | | 1357 (85.7) | |  |
| Family History of cancer | |  | | 0.116^a^ | |
| Yes | 451 (26.4) | | 380 (24.0) | |  |
| No | 1258 (73.6) | | 1203 (76.0) | |  |
| T category ^c^ |  | |  | | < 0.001^a^ |
| T1 | 87 (5.1) | | 91 (5.7) | |  |
| T2 | 92 (5.4) | | 101 (6.4) | |  |
| T3 | 1278 (74.8) | | 884 (55.8) | |  |
| T4 | 252 (14.7) | | 507 (32.1) | |  |
| N category ^c^ |  | |  | | < 0.001^a^ |
| N0 | 303 (17.7) | | 157 (9.9) | |  |
| N1 | 942 (55.1) | | 754 (47.6) | |  |
| N2 | 364 (21.3) | | 449 (28.4) | |  |
| N3 | 100 (5.9) | | 223 (14.1) | |  |
| Overall stage ^c^ |  | |  | | < 0.001^a^ |
| III | 1369 (80.1) | | 897 (56.7) | |  |
| IVA-B | 340 (19.9) | | 686 (43.3) | |  |
| LDH (U/L) |  | |  | | 0.320^b^ |
| Median (range) | 171 (67-564) | | 171 (39-626) | |  |

Abbreviations: NPC = nasopharyngeal carcinoma; CCRT = concurrent chemoradiotherapy; IC = induction chemotherapy; LDH = lactate dehydrogenase.

^a^ *P* values were calculated by Chi-square test.

^b^ *P* values were calculated by t test.

^c^ According to the 8th edition of UICC/AJCC staging system.
