# Supplementary material for: Individualized induction chemotherapy by pre-treatment plasma Epstein-Barr viral DNA in advanced nasopharyngeal carcinoma
Source: BMC Cancer. 2018 Dec 19;18:1276. doi: 10.1186/s12885-018-5177-9 (PMC6299978; doi:10.1186/s12885-018-5177-9)
Supplement: Supplementary file 5 — Table S3.Baseline characteristics of selected 1191 pairs with pre-treatment Epstein-Barr virus DNA ≤ 4650 copies/ml. (DOCX 16 kb) [file 12885_2018_5177_MOESM5_ESM.docx]

**Table S3**. Baseline characteristics of selected 1191 pairs with pre-treatment Epstein-Barr virus DNA ≤ 4650 copies/ml.

| Characteristics | CCRT (n=1191) | | IC+CCRT (n=1191) | | *P* value |
| --- | --- | --- | --- | --- | --- |
|  | No. (%) | | No. (%) | |  |
| Gender |  | |  | | 0.393^a^ |
| Female | 286 (24.0) | | 304 (25.5) | |  |
| Male | 905 (76.0) | | 887 (74.5) | |  |
| Age (years) |  | |  | | 0.375^b^ |
| Median (range) | 44 (18-76) | | 45 (18-75) | |  |
| Smoking |  | |  | | 0.609^a^ |
| Yes | 438 (36.8) | | 426 (35.8) | |  |
| No | 753 (63.2) | | 765 (64.2) | |  |
| Drinking |  | |  | | 1.000^a^ |
| Yes | 169 (14.2) | | 169 (14.2) | |  |
| No | 1022 (85.8) | | 1022 (85.8) | |  |
| Family History of cancer | |  | | 0.774^a^ | |
| Yes | 289 (24.3) | | 283 (23.8) | |  |
| No | 902 (75.7) | | 908 (76.2) | |  |
| T category ^c^ |  | |  | | 0.125^a^ |
| T1 | 58 (4.9) | | 78 (6.5) | |  |
| T2 | 73 (6.1) | | 73 (6.1) | |  |
| T3 | 811 (68.1) | | 764 (64.1) | |  |
| T4 | 249 (20.9) | | 276 (23.3) | |  |
| N category ^c^ |  | |  | | 0.170^a^ |
| N0 | 130 (10.9) | | 145 (12.3) | |  |
| N1 | 655 (55.0) | | 632 (53.1) | |  |
| N2 | 307 (25.8) | | 336 (28.2) | |  |
| N3 | 99 (8.3) | | 78 (6.4) | |  |
| Overall stage ^c^ |  | |  | | 0.785^a^ |
| III | 854 (71.7) | | 848 (71.2) | |  |
| IVA-B | 337 (28.3) | | 343 (28.8) | |  |
| LDH (U/L) |  | |  | | 0.171^b^ |
| Median (range) | 171 (67-564) | | 171 (39-353) | |  |

Abbreviations: NPC = nasopharyngeal carcinoma; CCRT = concurrent chemoradiotherapy; IC = induction chemotherapy; LDH = lactate dehydrogenase.

^a^ *P* values were calculated by Chi-square test.

^b^ *P* values were calculated by t test.

^c^ According to the 8th edition of UICC/AJCC staging system.
