# Supplementary material for: Individualized induction chemotherapy by pre-treatment plasma Epstein-Barr viral DNA in advanced nasopharyngeal carcinoma
Source: BMC Cancer. 2018 Dec 19;18:1276. doi: 10.1186/s12885-018-5177-9 (PMC6299978; doi:10.1186/s12885-018-5177-9)
Supplement: Supplementary file 6 — Table S4.Results of multivariate analysis for the selected 1191 pairs with pre-treatment Epstein-Barr virus DNA ≤ 4650 copies/ml. (DOCX 15 kb) [file 12885_2018_5177_MOESM6_ESM.docx]

**Table S4**. Results of multivariate analysis for the selected 1191 pairs with pre-treatment Epstein-Barr virus DNA ≤ 4650 copies/ml.

| Endpoints | Variable | HR (95% CI) | *P* value ^a^ |
| --- | --- | --- | --- |
| DFS | T category; T3-4 vs. T1-2 | 1.885 (1.284-2.766) | 0.001 |
|  | N category, N2-3 vs. N0-1 | 1.858 (1.472-2.345) | < 0.001 |
|  | Overall stage, IVA vs. III | 2.064 (1.665-2.559) | < 0.001 |
|  | Treatment, IC+CCRT vs. CCRT | 0.904 (0.730-1.119) | 0.354 |
| OS | T category; T3-4 vs. T1-2 | 1.892 (1.136-3.150) | 0.014 |
|  | N category, N2-3 vs. N0-1 | 2.213 (1.620-3.022) | < 0.001 |
|  | Overall stage, IVA vs. III | 2.763 (2.066-3.696) | < 0.001 |
|  | Treatment, IC+CCRT vs. CCRT | 1.012 (0.757-1.353) | 0.935 |
| DMFS | LDH; > 245 vs. ≤ 245 U/L | 2.124 (1.231-3.663) | 0.007 |
|  | T category; T3-4 vs. T1-2 | 1.949 (1.213-3.130) | 0.006 |
|  | N category, N2-3 vs. N0-1 | 2.623(1.941-3.546) | < 0.001 |
|  | Overall stage, IVA vs. III | 2.150 (1.620-2.855) | < 0.001 |
|  | Treatment, IC+CCRT vs. CCRT | 0.997 (0.752-1.322) | 0.986 |
| LRRFS | T category, T3-4 vs. T1-2 | 2.050 (1.135-3.703) | 0.017 |
|  | N category; N3 vs. N2 | 1.702 (1.210-2.395) | 0.002 |
|  | Overall stage, IVA vs. III | 1.980 (1.445-2.714) | < 0.001 |
|  | Treatment, IC+CCRT vs. CCRT | 0.952 (0.697-1.301) | 0.758 |

Abbreviations: DFS = disease-free survival; OS = overall survival; DMFS = distant metastasis-free survival; LRRFS = locoregional relapse-free survival; HR = hazard ratio; CI = confidence interval; IC = induction chemotherapy; CCRT = concurrent chemoradiotherapy; LDH = lactate dehydrogenase.

^a^ *P*-values were calculated using an adjusted Cox proportional hazards model with backward elimination and the following variables were included: gender (female vs. male), age (> 44y vs. ≤ 44y), smoking (yes vs. no), drinking (yes vs. no), family history of cancer (yes vs. no), LDH (> 245 vs. ≤ 245 U/L), T category (T3-4 vs. T1-2), N category (N2-3 vs. N0-1), overall stage (IVA vs. III) and treatment (IC+CCRT vs. CCRT).
