# Supplementary material for: Individualized induction chemotherapy by pre-treatment plasma Epstein-Barr viral DNA in advanced nasopharyngeal carcinoma
Source: BMC Cancer. 2018 Dec 19;18:1276. doi: 10.1186/s12885-018-5177-9 (PMC6299978; doi:10.1186/s12885-018-5177-9)
Supplement: Supplementary file 7 — Table S5.Baseline characteristics of 2926 patients with pre-treatment Epstein-Barr virus DNA > 4650 copies/ml. (DOCX 16 kb) [file 12885_2018_5177_MOESM7_ESM.docx]

**Table S5**. Baseline characteristics of 2926 patients with pre-treatment Epstein-Barr virus DNA > 4650 copies/ml.

| Characteristics | CCRT (n=999) | | IC+CCRT (n=1927) | | *P* value |
| --- | --- | --- | --- | --- | --- |
|  | No. (%) | | No. (%) | |  |
| Gender |  | |  | | 0.012^a^ |
| Female | 281 (28.1) | | 460 (23.9) | |  |
| Male | 718 (71.9) | | 1467 (76.1) | |  |
| Age (years) |  | |  | | 0.013^b^ |
| Median (range) | 46 (18-79) | | 44 (18-76) | |  |
| Smoking |  | |  | | 0.009^a^ |
| Yes | 359 (35.9) | | 789 (40.9) | |  |
| No | 640 (64.1) | | 1138 (59.1) | |  |
| Drinking |  | |  | | 0.245^a^ |
| Yes | 163 (16.3) | | 283 (14.7) | |  |
| No | 836 (83.7) | | 1644 (85.3) | |  |
| Family History of cancer | |  | | 0.497^a^ | |
| Yes | 239 (23.9) | | 483 (25.1) | |  |
| No | 760 (76.1) | | 1444 (74.9) | |  |
| T category ^c^ |  | |  | | < 0.001^a^ |
| T1 | 53 (5.3) | | 88 (4.6) | |  |
| T2 | 97 (9.7) | | 171 (8.9) | |  |
| T3 | 607 (60.8) | | 960 (49.8) | |  |
| T4 | 242 (24.2) | | 708 (36.7) | |  |
| N category ^c^ |  | |  | | < 0.001^a^ |
| N0 | 51 (5.2) | | 56 (2.9) | |  |
| N1 | 458 (45.8) | | 715 (37.1) | |  |
| N2 | 320 (32.0) | | 630 (32.7) | |  |
| N3 | 170 (17.0) | | 526 (27.3) | |  |
| Overall stage ^c^ |  | |  | | < 0.001^a^ |
| III | 613 (61.4) | | 809 (42.0) | |  |
| IVA-B | 386 (38.6) | | 1118 (58.0) | |  |
| LDH (U/L) |  | |  | | 0.001^b^ |
| Median (range) | 181 (95-1009) | | 188 (93-774) | |  |

Abbreviations: NPC = nasopharyngeal carcinoma; CCRT = concurrent chemoradiotherapy; IC = induction chemotherapy; LDH = lactate dehydrogenase.

^a^ *P* values were calculated by Chi-square test.

^b^ *P* values were calculated by t test.

^c^ According to the 8th edition of UICC/AJCC staging system.
