# Supplementary material for: Individualized induction chemotherapy by pre-treatment plasma Epstein-Barr viral DNA in advanced nasopharyngeal carcinoma
Source: BMC Cancer. 2018 Dec 19;18:1276. doi: 10.1186/s12885-018-5177-9 (PMC6299978; doi:10.1186/s12885-018-5177-9)
Supplement: Supplementary file 8 — Table S6.Baseline characteristics of selected 945 pairs with pre-treatment Epstein-Barr virus DNA > 4650 copies/ml. (DOCX 16 kb) [file 12885_2018_5177_MOESM8_ESM.docx]

**Table S6**. Baseline characteristics of selected 945 pairs with pre-treatment Epstein-Barr virus DNA > 4650 copies/ml.

| Characteristics | CCRT (n=945) | | IC+CCRT (n=945) | | *P* value |
| --- | --- | --- | --- | --- | --- |
|  | No. (%) | | No. (%) | |  |
| Gender |  | |  | | 0.356^a^ |
| Female | 254 (26.9) | | 272 (28.8) | |  |
| Male | 691 (73.1) | | 673 (71.2) | |  |
| Age (years) |  | |  | | 0.469^b^ |
| Median (range) | 45 (18-77) | | 46 (18-74) | |  |
| Smoking |  | |  | | 0.250^a^ |
| Yes | 351 (37.1) | | 327 (34.6) | |  |
| No | 594 (62.9) | | 618 (65.4) | |  |
| Drinking |  | |  | | 0.899^a^ |
| Yes | 149 (15.8) | | 147 (15.6) | |  |
| No | 796 (84.2) | | 798 (84.4) | |  |
| Family History of cancer | |  | | 0.629^a^ | |
| Yes | 225 (23.8) | | 234 (24.8) | |  |
| No | 720 (76.2) | | 711 (75.2) | |  |
| T category ^c^ |  | |  | | 0.449^a^ |
| T1 | 44 (4.7) | | 60 (6.4) | |  |
| T2 | 93 (9.8) | | 94 (9.9) | |  |
| T3 | 566 (59.9) | | 554 (58.6) | |  |
| T4 | 242 (25.6) | | 237 (25.1) | |  |
| N category ^c^ |  | |  | | 0.321^a^ |
| N0 | 37 (3.9) | | 32 (3.4) | |  |
| N1 | 432 (45.7) | | 435 (46.1) | |  |
| N2 | 306 (32.4) | | 333 (35.2) | |  |
| N3 | 170 (18.0) | | 145 (15.3) | |  |
| Overall stage ^c^ |  | |  | | 0.347^a^ |
| III | 559 (59.2) | | 579 (61.3) | |  |
| IVA-B | 386 (40.8) | | 366 (38.7) | |  |
| LDH (U/L) |  | |  | | 0.720^b^ |
| Median (range) | 181 (95-1009) | | 186 (93-668) | |  |

Abbreviations: NPC = nasopharyngeal carcinoma; CCRT = concurrent chemoradiotherapy; IC = induction chemotherapy; LDH = lactate dehydrogenase.

^a^ *P* values were calculated by Chi-square test.

^b^ *P* values were calculated by t test.

^c^ According to the 8th edition of UICC/AJCC staging system.
